# Feasibility of Human Neural Stem Cell Transplantation for the Treatment of Acute Subdural Hematoma in a Rat Model: A Pilot Study

**DOI:** 10.3389/fneur.2019.00082

**Published:** 2019-02-12

**Authors:** Shoji Yokobori, Kazuma Sasaki, Takahiro Kanaya, Yutaka Igarashi, Ryuta Nakae, Hidetaka Onda, Tomohiko Masuno, Satoshi Suda, Kota Sowa, Masataka Nakajima, Markus S. Spurlock, Lee Onn Chieng, Tom G. Hazel, Karl Johe, Shyam Gajavelli, Akira Fuse, M. Ross Bullock, Hiroyuki Yokota

**Affiliations:** ^1^Department of Emergency and Critical Care Medicine, Nippon Medical School, Tokyo, Japan; ^2^Department of Neurological Science, Graduate School of Medicine, Nippon Medical School, Tokyo, Japan; ^3^Department of Neurosurgery, University of Miami Miller School of Medicine, Miami, FL, United States; ^4^Neuralstem, Inc., Germantown, MD, United States

**Keywords:** traumatic brain injury, acute subdural hematoma, transplantation, neural stem cell, treatment

## Abstract

Human neural stem cells (hNSCs) transplantation in several brain injury models has established their therapeutic potential. However, the feasibility of hNSCs transplantation is still not clear for acute subdural hematoma (ASDH) brain injury that needs external decompression. Thus, the aim of this pilot study was to test feasibility using a rat ASDH decompression model with two clinically relevant transplantation methods. Two different methods, *in situ* stereotactic injection and hNSC-embedded matrix seating on the brain surface, were attempted. Athymic rats were randomized to uninjured or ASDH groups (F344/NJcl-rnu/rnu, *n* = 7–10/group). Animals in injury group were subjected to ASDH, and received decompressive craniectomy and 1-week after decompression surgery were transplanted with green fluorescent protein (GFP)-transduced hNSCs using one of two approaches. Histopathological examinations at 4 and 8 weeks showed that the GFP-positive hNSCs survived in injured brain tissue, extended neurite-like projections resembling neural dendrites. The *in situ* transplantation group had greater engraftment of hNSCs than matrix embedding approach. Immunohistochemistry with doublecortin, NeuN, and GFAP at 8 weeks after transplantation showed that transplanted hNSCs remained as immature neurons and did not differentiate toward to glial cell lines. Motor function was assessed with rotarod, compared to control group (*n* = 10). The latency to fall from the rotarod in hNSC *in situ* transplanted rats was significantly higher than in control rats (median, 113 s in hNSC vs. 69 s in control, *P* = 0.02). This study first demonstrates the robust engraftment of *in situ* transplanted hNSCs in a clinically-relevant ASDH decompression rat model. Further preclinical studies with longer study duration are warranted to verify the effectiveness of hNSC transplantation in amelioration of TBI induced deficits.

## Introduction

Despite much effort, the prognosis of acute subdural hematoma (ASDH) remains quite poor (1). In the US, approximately 50,000 people die and at least 5.3 million live with disabilities related to traumatic brain injury (TBI) per year, with ASDH significantly contributing to these (2, 3). Further, ASDH is recognized as a leading cause of mortality and morbidity in young adults globally (4–6), while elderly patients with ASDH have the worst outcomes among those with other types of severe TBI (5–7). Thus, ASDH constitutes a heavy burden both on patients and their families.

The pathology of severe TBI has been classically divided as primary and secondary brain injury. Primary brain injury is defined as the one occurring immediately after receiving external force to the cranium. In contrast, secondary brain injury is the additional injury caused by aggravating extracranial and intracranial factors, including hypoxia, hypotension, and hypoperfusion. Since primary injury is unavoidable, the aim of current treatments has been to diminish secondary brain injury. However, several clinical randomized controlled trials have not been able to show efficient reduction in patients' mortality (3, 7–9). The treatment strategies have only focused on preventing secondary brain injury and thus have been limited in clinical situations. Therefore, novel approaches, like regenerating medicine or cell-therapy are needed (10, 11).

In the past decade, a commercially available human neural stem cell (hNSC) line, NSI-566 cells, derived from human 8-week-old fetal spinal cord, has been established for cell-therapy (12). This cell line has been already approved to use in human clinical trials by the US Food and Drug Administration. A recent study showed efficient hNSC transplantation for the amelioration of cognitive function in a rat model of penetrating ballistic-like brain injury (13), as well as in murine models of cortical TBI (14, 15). However, the use of regenerating treatment for more common type TBI (i.e., ASDH) models has not been attempted until now. The aim of this pilot study was to assess the feasibility of hNSC transplantation in a clinically-relevant model of ASDH/decompression in rats.

## Materials and Methods

### Study Design and Animals

Procedures for all animal experiments followed the guidelines established by the Japanese Ministry of Education, Culture, Sports, Science, and Technology Guide for the Care and Use of Laboratory Animals and Animal Research. The protocol of the study was approved by the Nippon Medical School's Institutional Animal Care and Use Committees (NMS IACUC #28-044).

Surgical procedures were performed under aseptic conditions. Athymic rats (F344/NJcl-rnu/rnu, male, 200 g) were used in this study.

#### Anesthesia

Anesthesia was induced with isoflurane (1–2%) delivered in a mixture with 30% oxygen. Body temperature was maintained at normothermia (36 ± 0.5°C) throughout all surgical procedures using a homeothermic heating pad (Harvard Apparatus, South Natick, MA). The tail artery was cannulated with a PE-50 tube in each rat for blood pressure measurement, arterial blood gas monitoring, and for drawing autologous blood for ASDH induction. The pH, PaCO_2_, and PaO_2_ were measured using a portable blood gas analyzer (i-STAT 1 analyzer, Abbott Point of Care Inc., Princeton, NJ) before and after ASDH induction.

#### Induction of Subdural Hematoma

**Figure 1:**
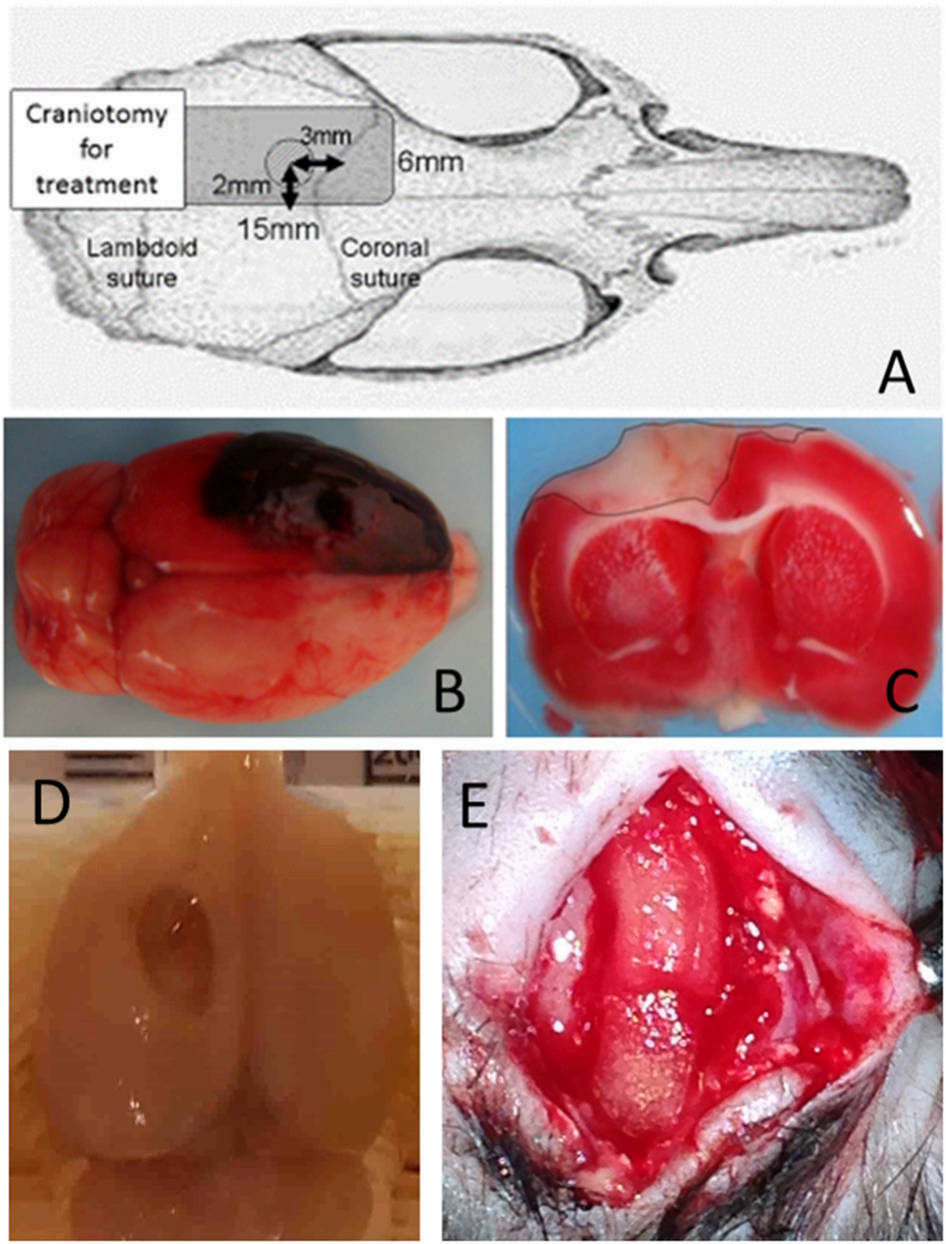
Schematic illustration and confirmation of ASDH model. **(A)** Schematic illustration of subdural hematoma induction. **(B)** ASDH was induced by injecting autologous blood, allowing it to clot *in situ*. To mimic clinical conditions, hemicraniectomy for hematoma removal and decompression were performed after ASDH induction. **(C)** Triphenyltetrazolium chloride staining showing the injured cortical area and verifying the successful generation of the ASDH model. **(D)** Macroscopic injury findings 8weeks after ASDH induction. **(E)** Superficial placement of NSI-566 embedded artificial dura (DuraGen), with mimicking dural plasty in clinical situation (dotted rectangles).

Details for the induction of subdural hematoma and performance of the decompression surgery have been described in our previous reports (16–20). Briefly, the scalp was incised on the midline, and a single burr hole of 3 mm in diameter was drilled 2 mm to the left of the sagittal suture and 3 mm behind the coronal suture (Figure 1A). The dura was then incised under a microscope, and a blunt-tipped PE-50 polyethylene tube was inserted into the subdural space. Quick-setting cyanoacrylate glue was used to fix the tube. The burr hole was then sealed with dental cement. The hematoma was induced with the injection of non-heparinized autologous blood (250 μL) into the subdural space over 5 min, allowing it to clot *in situ* (Figure 1B). After injection, the induction tube was cut off and sealed.

Thirty minutes after induction of the hematoma, a rectangular craniotomy (15 × 6 mm) was performed using a dental drill (Figure 1A). The subdural hematoma was removed by saline irrigation and forceps, after widely opening the dura. The scalp was then closed over the craniotomy window without replacing the bone, to mimic the clinical practice of decompressive craniotomy. Thus, the ischemic/reperfusion TBI model with large cortical injury (~6 mm^2^) was created (Figure 1C; seen by triphenyltetrazolium chloride staining, and Figure 1D; macroscopic findings) (21).

#### Cell Transplantation

hNSC transplantation was performed 7 days after the surgical decompression treatment. Two different methods for the transplantation were attempted as below. In both of transplantation method, rats were anesthetized with 2% isoflurane and secured in a stereotaxic frame; the scalp was reopened along the midline to expose the injured cortical surface.

1. hNSC *in situ* transplantation
  A microsyringe was backfilled and flushed with suspension media, then attached to a microsyringe injector and micro4 controller (UMP3-3, World Precision Instruments, Sarasota, FL). The microsyringe was then filled with green fluorescent protein (GFP)-transduced hNSC cells (NSI-566, Neuralstem, Inc. Germantown, Maryland, USA) in suspension media (in a concentration of 100,000 cells/μL) (22). The cell density was certified by cell counting with 0.4% Trypan blue solution and hemocytometer. The injection was administered at −3 mm AP and +2 mm ML from the bregma, ipsilateral to the injury, targeted proximal to the injured motor cortical area. The microsyringe was advanced vertically 4-mm deep into the brain. Using the micro pump, 2 μL were injected at a rate of 1 μL per min. The needle was then retracted from the brain. In total, 2 × 10^5^ cells were transplanted in the injured cortex.
2. hNSC transplantation on the cortical surface
  For this method, a bovine tendon derived collagen-based dural regeneration matrix (DuraGen, Integra, NJ, USA) was applied. On this matrix, hNSCs (in a concentration of 100,000 cells/μL) were embedded. After reopened the scalp, embedded matrix was seated on the injured cortical surface mimicking duralplasty in clinical situation (Figure 1E). The scalp was then re-sutured with aseptic condition.

### Behavioral Testing for Assessing Motor Function

Motor function and its recovery were assessed every week for 4 weeks after transplantation using the rotarod performance test (23). The latency to fall from the rotarod was scored automatically with infrared sensors in Rotamex 5 rotarod (Columbus Inst, Columbus, OH, USA). Each week, three trials were performed for each rat (23, 24), and the best score was retained for the analysis. The acceleration step and time were determined empirically. The speed was increased by 0.5 cm/s every 5 s.

### Specimen Collection, Histology, and Imaging

Four to eight weeks after transplantation, rats were transcardially perfused with 0.1 M phosphate buffered saline, followed by cold 4% paraformaldehyde in 0.1 M phosphate buffer. Brains were dissected (Figure 1D) and post-fixed in the same solution for 12 h and then transferred to a 30% sucrose solution for 24 h. Brains were frozen in embedding matrix using dry ice and stored at −20°C before being sectioned on a cryostat at 40-μm thickness. Free-floating sections were stored in 0.02% sodium aside in phosphate buffered saline prior to immunohistochemistry. Samples were stained with 4′,6-diamidino-2-phenylindole (DAPI) to mark neuronal nuclei and GFP to confirm the presence of transplanted hNSCs. Samples were also assessed with the following primary antibodies: NeuN (Millipore MAB377), DCX (Millipore AB2253), GFAP (Dako Z0334), and IBA-1(Millipore MABN92). Fluorescent images were observed on a confocal microscope (OLYMPUS BX51, Olympus Optical Co., Ltd., Tokyo, Japan).

### Statistical Analysis

Non-parametric data were compared using the Mann–Whitney *U*-test. All non-parametric data are presented as the median and interquartile range (IQR). Rotarod results were compared using two-way repeated measures analysis of variance with Fisher's least significant difference *post-hoc* method. All analyses were performed using StatFlex software (version 6.0; Artech Co. Ltd., Osaka, Japan), and differences were considered statistically significant at a *P* < 0.05.

## Results

### Baseline Characteristics

We did not find any significant differences between the all transplanted groups and control groups on body weight, body temperature, mean arterial pressure, pH, PaO_2_, and PaCO_2_ before and after ASDH induction. Changes in body weights during study period were not significantly different among all treatment groups. All rats, in all groups, survived for at least 4 weeks after injury and craniotomy.

### Histological Analysis

**Figure 2:**
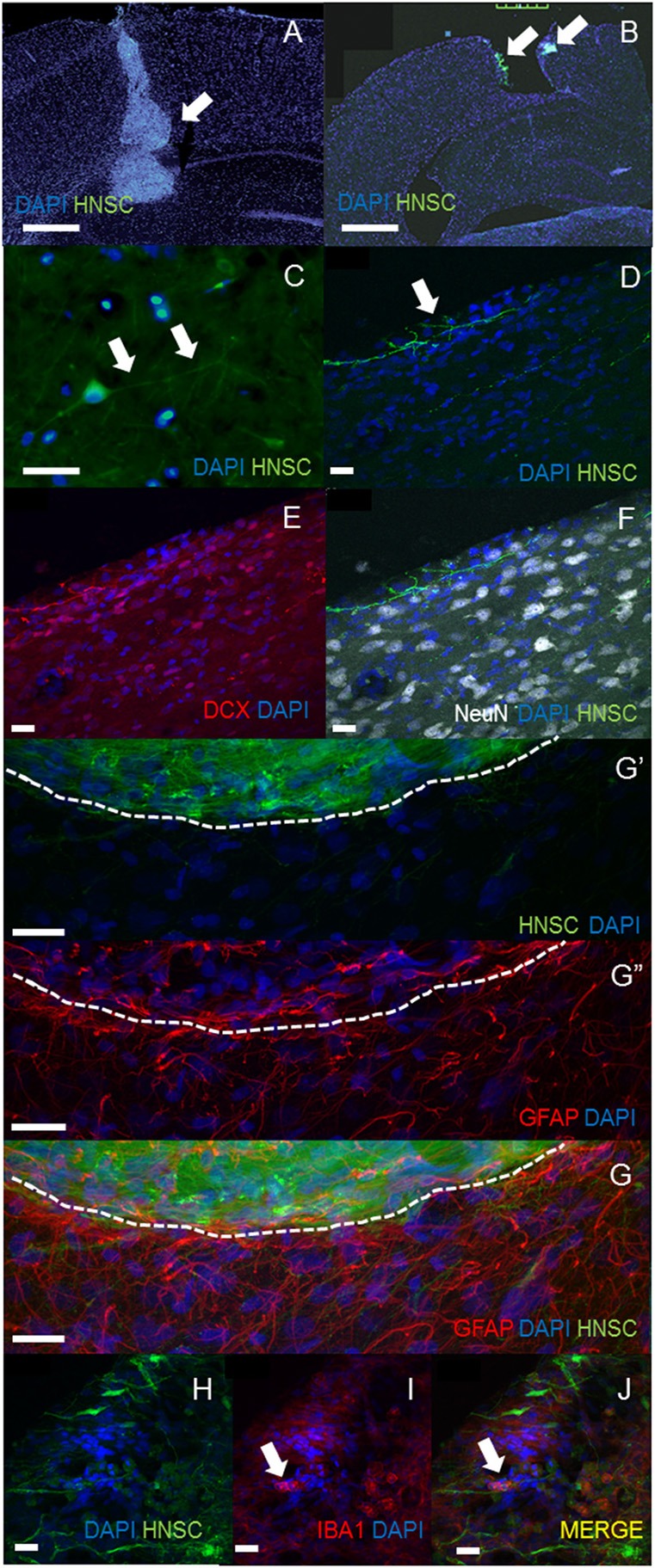
Histological analysis. The engraftment of NSCs could be seen in the injured cortex in *in situ* transplantation **(A)** and the surface of cortex in superficial transplantation **(B)** at least 5 weeks post-transplantation (**A,B**, scale bar = 100 μm). Much robust engraftment of hNSCs were observed in *in situ* transplanted groups **(A)**. Higher magnification of GFP-positive transplanted hNSCs revealed long processes, resembling neurites [white arrows, **(C)** in *in situ* transplantation, scale bar = 40 μm; **(D)** in surface seating transplantation] and extending across the injured motor cortex. These cells had firm nuclear and neurites structure which was stained by DCX but not stained by NeuN (**D–F**, scale bar = 20 μm). Confocal images of brain sections stained with anti-GFAP antibody also showed absence of GFAP expression in transplanted human hNSC but presence of gliosis at the host-transplant border (dashed white line **G,G′,G′′**, scale bar = 20 μm). Iba-1, a phagocytic markers identifies microglia/infiltrating immune cells (white arrow in **H–J**, scale bar = 20 μm).

In both of transplantation method, the engraftment of NSCs could be seen in the injured cortex and the surface of cortex at least 5 weeks post-transplantation (Figures 2A,B). Several transplanted GFP positive NSI-566 hNSC cells were present in the injured cortex and the hippocampus of the ipsilateral side. However, much robust engraftment of hNSCs were observed in *in situ* transplanted groups. Higher magnification of GFP-positive transplanted hNSCs revealed long processes, resembling neurites (white arrows, Figure 2C in *in situ* transplantation, Figure 2D in superficial seating transplantation) and extending across the injured motor cortex. These cells had firm nuclear and neurites structure which was stained by DCX but not stained by NeuN (Figures 2E,F). Confocal images of brains sections stained with anti-GFAP antibody also showed absence of GFAP expression in transplanted human hNSC but presence of gliosis at the host-transplant border (dashed white line in Figure 2G). Iba-1, a phagocytic markers identifies microglia/infiltrating immune cells (white arrow in Figures 2H–J).

### Rotarod Performance Testing for Motor Function

**Figure 3:**
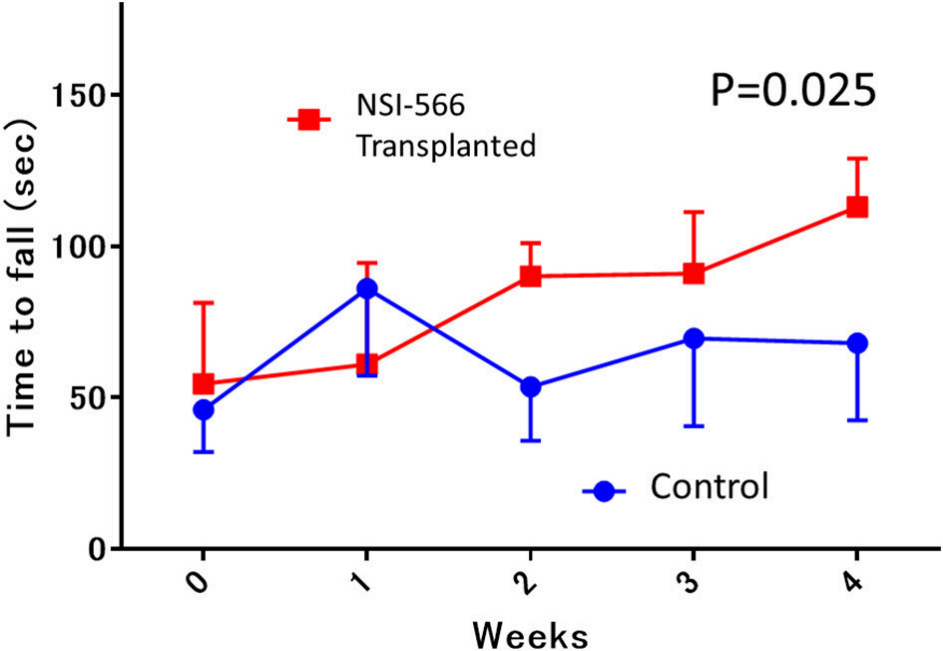
Rotarod motor performance testing. In the 4th week after transplantation, the median latency to fall from the rotarod in the human neural stem cell-transplanted rats was significantly longer (red line; NSI-566) than in control rats (blue line), Data are shown as median and interquartile range.

Rotarod performance testing for motor function was performed in *in situ* transplanted rats (TP group vs. Control group, *n* = 10 each). The median latency to fall from the rotarod in the TP group was significantly superior to that in the control group (median [IQR]: 113 s [76–121] in TP vs. 69 s [43–69] in control, ANOVA, *P* = 0.025; Figure 3).

## Discussion

This is the first study demonstrating the feasibility of hNSC transplantation in a clinically-relevant ASDH decompression rat model. In this pilot trial, we found that the transplanted hNSCs were robustly engrafted even until the fourth week after transplantation. GFP-positive hNSCs extended projections resembling neural dendrites. All hNSC transplanted cells were immature but had a tendency to mature toward neuronal cell lines. Rats transplanted with hNSCs survived without weight loss or any adverse effect, similar to rats in the control group. They seemed to perform better behavioral outcome on the rotarod test to control rats.

For the treatment of ASDH associated with intracranial hypertension and cerebral herniation, surgery with craniotomy, to achieve mass evacuation, is recommended as the standard treatment (25). However, the removal of the subdural hematoma itself results in the immediate reversal of global ischemia and the induction of reperfusion injury (16, 26). Previous experimental and clinical studies have clearly shown that the pathophysiology of ASDH and its removal are synonymous with “ischemic/reperfusion injury” (17, 27). Thus, the pathophysiology of ASDH treated with decompressive craniotomy is quite unique compared to other types of TBI.

To improve the outcome in such patients, several clinical randomized controlled trials have been performed. Recently, the RESCUE-ICP trial clarified that decompressive craniotomy can improve patients' survival after surgical decompression; however, this trial also demonstrated an increased rate of worse functional outcomes, i.e., persistent vegetable state and severe disability (26). Thus, to improve the functional sequelae in these patients, the novel approach of regenerative medicine is needed.

To date, several preclinical studies have evaluated the efficacy of rodent neural precursor cell transplantation in rodent TBI models (28). The most promising results have been produced by using immunodeficient rodents or host immunosuppression and same-species allografting (29, 30). The exogenous NSCs have been found to integrate into the rodent injured host, aiding endogenous repair and modifying behavior (13).

To study the feasibility of cell transplantation therapy in this unique pathophysiology of ischemic/reperfusion brain injury, we used an ASDH/decompressive craniotomy rat model. We believe that this model shares some characteristics with the simple type ASDH pathophysiology, caused by the slow accumulation of hemorrhage in the subdural space, thus mimicking the clinical disruption of small bridging vessels. Most elderly patients with ASDH have this simple type pathophysiology due to low-impact injuries, such as those caused by tumbling. In Japan, this type of ASDH is actually increasing with the increase in longevity (31). To reflect real-world situations, we considered this model as suitable for our pilot study.

In this pilot study, hNSCs survived and integrated in the ischemic reperfused brain, after surgical decompression. Additionally, transplanted cell morphology changed such that they extended long processes like neurites in the injured cortex and hippocampus. Our IHC data also showed that these hNSCs did not mature but differentiated toward neuronal lineage. Moreover, hNSC transplantation seemed to have better motor functional capacity.

Recently, Inoue et al. showed that rotarod motor performance recovers with the expression of brain derived neurotrophic factor in the motor cortex (32). In our experiment, hNSCs seemed not to be matured yet within 4–8 weeks after transplantation, therefore, in this phase, NSI-566 transplantation may have a potential to support neuronal recovery through secreting neurotrophic factors rather than replenishing neural networks in the motor cortex. Further pathophysiological and electrophysiological examinations with long durations are required to validate our findings.

Our study also demonstrated the safety of hNSC transplantation. Even in immunosuppressed rats, there were no differences in mortality or any side effects compared to control rats. The median body weight did not differ between the TP and control groups, and the transplanted hNSCs did not show malignant proliferation for up to 8 weeks after transplantation. All rats survived in both groups. These results may serve as a basis for further preclinical animal experiments and clinical trials in the near future.

### Limitations

This study has several limitations. First, we used immunosuppressed athymic rats. For translating the results into clinical practice, we should apply this approach to healthy rats without immunosuppression. For this purpose, an appropriate regimen for immunosuppression should be considered. According to a recent publication, intra-peritoneal tacrolimus, methylprednisolone, and mycophenolate mofetil injection were effective for the robust engraftment of hNSCs in a rat model (33). The safety of this regimen should also be confirmed in the ASDH rat model.

Second, we only tracked the engrafted hNSCs for 4–8 weeks after transplantation. In future studies, this observation period will be prolonged. In our recent study, in which we transplanted hNSCs in a rat model of penetrating ballistic-like brain injury, we showed that transplanted cells can survive for up to 16 weeks (13). For the translation of our findings to clinical situations, longer safety and feasibility studies need to be performed.

Third, we only estimated motor function recovery. According to previous literature (14), we considered that rotarod was suitable for motor function testing in the NSC-transplanted animals. To confirm the recovery of spatial learning and memory, other tests should be performed in future studies, like the Y-maze or Morris water maze.

Fourth, our study design did not include sham or naïve surgery. However, since our aim was to clarify the feasibility of NSC transplantation, we only compared injured rats with or without transplantation. For higher accuracy, sham or naïve operated animals should also be examined. These limitations should be taken into consideration in further preclinical studies.

## Conclusion

In conclusion, our pilot study provides evidence for the feasibility and safety of hNSCs in a rat model of clinical ASDH/decompression. For clinical translatability, future large-scale preclinical studies are warranted.

## Data Availability Statement

The raw data supporting the conclusions of this manuscript will be made available by the authors to any qualified researcher.

## Author Contributions

SY, MS, SG, and MB: conception and design. SY and SS: analysis and interpretation of data. SY drafting the article. All authors: critically revising the article. All authors reviewed submitted version of manuscript. SY approved the final version of the manuscript on behalf of all authors. TH and KJ: administrative/technical/material support. MB and HY: study supervision.

### Conflict of Interest Statement

TH and KJ are employees of Neuralstem, Inc. The remaining authors declare that the research was conducted in the absence of any commercial or financial relationships that could be construed as a potential conflict of interest.

## Abbreviations

ASDH: acute subdural hematoma
DAPI, 4': 6-diamidino-2-phenylindole
GFP: green fluorescent protein
hNSCs: human neural stem cells
IQR: interquartile range
TBI: traumatic brain injury
TP: transplantation.
